# A Deep Learning Pipeline for Grade Groups Classification Using Digitized Prostate Biopsy Specimens

**DOI:** 10.3390/s21206708

**Published:** 2021-10-09

**Authors:** Kamal Hammouda, Fahmi Khalifa, Moumen El-Melegy, Mohamed Ghazal, Hanan E. Darwish, Mohamed Abou El-Ghar, Ayman El-Baz

**Affiliations:** 1 BioImaging Laboratory, Bioengineering Department, University of Louisville, Louisville, KY 40292, USA; kamal.hammouda@louisville.edu (K.H.); fahmi.khalifa@louisville.edu (F.K.); 2 Department of Electrical Engineering, Assiut University, Assiut 71515, Egypt; moumen@aun.edu.eg; 3 Electrical and Computer Engineering Department, Abu Dhabi University, Abu Dhabi 59911, United Arab Emirates; mohammed.ghazal@adu.ac.ae; 4 Mathematics Department, Faculty of Science, Mansoura University, Mansoura 35516, Egypt; Hedarwish@mans.edu.eg; 5 Radiology Department, Urology and Nephrology Center, Mansoura University, Mansoura 35516, Egypt; maboelghar@mans.edu.eg

**Keywords:** deep learning, classification, grade groups, CAD system, prostate cancer

## Abstract

Prostate cancer is a significant cause of morbidity and mortality in the USA. In this paper, we develop a computer-aided diagnostic (CAD) system for automated grade groups (GG) classification using digitized prostate biopsy specimens (PBSs). Our CAD system aims to firstly classify the Gleason pattern (GP), and then identifies the Gleason score (GS) and GG. The GP classification pipeline is based on a pyramidal deep learning system that utilizes three convolution neural networks (CNN) to produce both patch- and pixel-wise classifications. The analysis starts with sequential preprocessing steps that include a histogram equalization step to adjust intensity values, followed by a PBSs’ edge enhancement. The digitized PBSs are then divided into overlapping patches with the three sizes: 100 × 100 ($CNN_{S}$), 150 × 150 ($CNN_{M}$), and 200 × 200 ($CNN_{L}$), pixels, and 75% overlap. Those three sizes of patches represent the three pyramidal levels. This pyramidal technique allows us to extract rich information, such as that the larger patches give more global information, while the small patches provide local details. After that, the patch-wise technique assigns each overlapped patch a label as GP categories (1 to 5). Then, the majority voting is the core approach for getting the pixel-wise classification that is used to get a single label for each overlapped pixel. The results after applying those techniques are three images of the same size as the original, and each pixel has a single label. We utilized the majority voting technique again on those three images to obtain only one. The proposed framework is trained, validated, and tested on 608 whole slide images (WSIs) of the digitized PBSs. The overall diagnostic accuracy is evaluated using several metrics: precision, recall, F1-score, accuracy, macro-averaged, and weighted-averaged. The ($CNN_{L}$) has the best accuracy results for patch classification among the three CNNs, and its classification accuracy is 0.76. The macro-averaged and weighted-average metrics are found to be around 0.70–0.77. For GG, our CAD results are about 80% for precision, and between 60% to 80% for recall and F1-score, respectively. Also, it is around 94% for accuracy and NPV. To highlight our CAD systems’ results, we used the standard ResNet50 and VGG-16 to compare our CNN’s patch-wise classification results. As well, we compared the GG’s results with that of the previous work.

## 1. Introduction

The most recent statistics from the American Cancer Society showed that prostate cancer (PC) is the most prevalent type of cancer with 248,530 (26%) cases, and it is also the second leading cause of cancer-related death with 34,130 (26%) [1] among men. Prostate tumors are like many other cancers in that the initial stage does not cause death or pain. By time the tumor is recognized, it has advanced to high grade with increase mortality [1]. The pathological evaluation of prostate biopsies determines the best treatment method of PC [2]. One of the methods used to characterize the heterogeneous tumor growth patterns is the Gleason grading system, which observes in a biopsy regarding their degree of discrimination or the Gleason pattern (GP).

The GP practically ranges from GP1 through GP5. The GP1 (stroma) and GP2 (benign) represent the nonepithelium tissue. While GP3, GP4, and GP5 represent the epithelium tissue. GP3 indicates moderately differentiated glands compared with that of GP5, which represents poorly differentiated cells [3,4]. Many factors contribute to determining the stage of PC, like the prostate-specific antigen (PSA) level. However, the primary factor is the Gleason score (GS). The GS is the grading system used to determine PC’s aggressiveness depending on the two most frequent GP observed in the biopsy [5]. Typically, the GS ranges from 6 to 10, where 6 illustrates low-grade cancer, i.e., the cancer is likely to grow slowly, and 10 represents high-grade, i.e., the cancer is expected to spread more rapidly.

**Figure 1:**
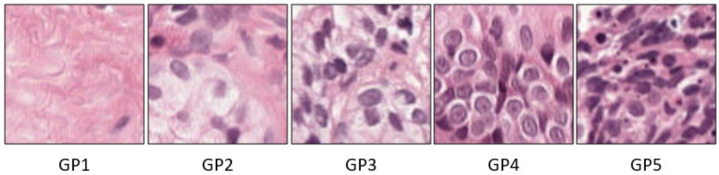
Examples of GP labels.

The GS grading system is often divided into only three categories, 6, 7, and 8–10 [5]. This classification is rather coarse. For example, GS7 could indicate that the most cells are GP3, followed by GP4, or that most cells are GP4, followed by GP3; however, the latter case has a much worse prognosis. Similarly, GS9 or GS10 has a worse prognosis than GS8 despite often being grouped together. Eventually, the 2014 International Society of Urological Pathology (ISUP) developed a simple grading system for PC: grade groups (GG) system, based on the visual assessment of cell differentiation and GP predominance [6], see Figure 1. The GG ranges from GG1 to GG5, with higher GG indicating greater clinical risk. Table 1 shows the relation between the Gleason grading (GP and GS) and the GG system, besides the shape of cell tissues for GP and the GG’s risk level.

The discordance in GG diagnosis by different pathologists using the same biopsy is between 30–50% [3,7,8]. Agreement is greater among pathologists with urologic subspecialty training and high experience than among pathologists in general [9]. Accurate diagnosis of prostate biopsy specimens helps physicians to make essential treatment decisions [9,10]. Due to expert subspecialists’ availability, the development of an automated system for assessing prostate biopsy specimens with expert-level performance could improve prostate biopsy’s clinical utility.

In recent years, extensive research work was developed to diagnose the tumorous lesions for many organs, especially the prostate [11,12,13,14]. Deep learning (DL) combined with histopathology and radiology imaging, particularly magnetic resonance imaging (MRI), plays an essential role in grading the prostate’s cancerous tissues [4,15,16,17]. For histopathology, Arvaniti et al. [18] developed a DL approach to identify automated Gleason grading of prostate cancer tissue microarrays with Hematoxylin and Eosin (H&E) staining. This model’s advantages were that it was trained by a dataset of about 641 patients and tested on an independent cohort of 245 patients annotated by two pathologists. Cohen’s quadratic kappa statistic was used to the interannotator agreements between the model and each pathologist. The authors reported the results for the GP and GS. However, they did not mention the final classification of the GG that is considered the simple grading system for PC. Also, Bulten et al. [19] introduced automated grading prostate biopsies using a DL system. They focus on classifying the GP for the prostate biopsies, and then identifying the GS depending on the GP predominance. Their DL approach was developed using 5834 biopsies from 1243 patients. The authors did not report the overall accuracy of the GP classification; they reported only the final results for the GG. Similarly, Nagpal et al. [4] used a DL approach to improve GS for whole-slide images of prostatectomies. The system was developed using 112 million pathologist-annotated image patches from 1226 slides and tested on an independent validation dataset of 331 slides. The author considered the GG4 with GG5 as one class and did not report the individual results for both of them. The view tissue for the GG5 is very similar to the GG4, and it is considered the big challenge to differentiate between them.

According to the previous studies, the general technique to classify the GG, (see e.g., the work in [19]) is that the DL systems are developed to segment digitized prostate biopsy specimens (PBSs) into regions according to GP, after which the GG is identified depending on the GS and GP grade and its predominance. However, no study reported the overall accuracy of the GP segmentation. They reported only the final results for the GG. Our work develops a DL-based computer-aided diagnostic (CAD) system for reading digitized PBSs sections and dealing with GP as a classification problem, not as a segmentation task using patch- and pixel-wise classification methodology. This is the first time this ideas was applied, to the best of our knowledge. Finally, GP labels are used to determine the GS and GG, and their performance is comparable to expert subspecialists.

The rest of the paper is structured as follows. Section 2 describes in details the proposed DL pipeline. The performance metrics used for evaluation and the details of experimental results are given in Section 3. The limitation and highlights for our pipeline are discussed in Section 4. Finally, Section 5 concludes the work.

## 2. Methods

**Figure 2:**
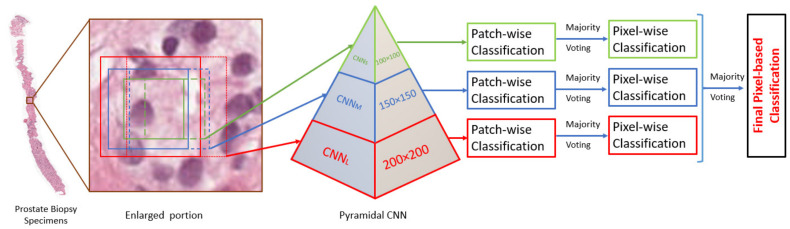
The proposed pipeline for our CAD system.

This work’s primary objective is to develop a CAD system for accurate GG diagnosis of PBSs. The proposed overall CAD system performs GP classification, as well as identifies the GS and GG. The GP classification pipeline consists of three stages: a DL system consisting of fusion three convolution neural networks (CNN); namely, a pyramidal CNN. Also, a patch and pixel-wise classification that divided the original image into patches and labeled them according to GP, the majority voting techniques is used in this step to merge the patches images into the original size, see Figure 2. Finally, identifying the GS and GG depends on the classification of the GP.

### 2.1. Deep Learning System

**Figure 3:**
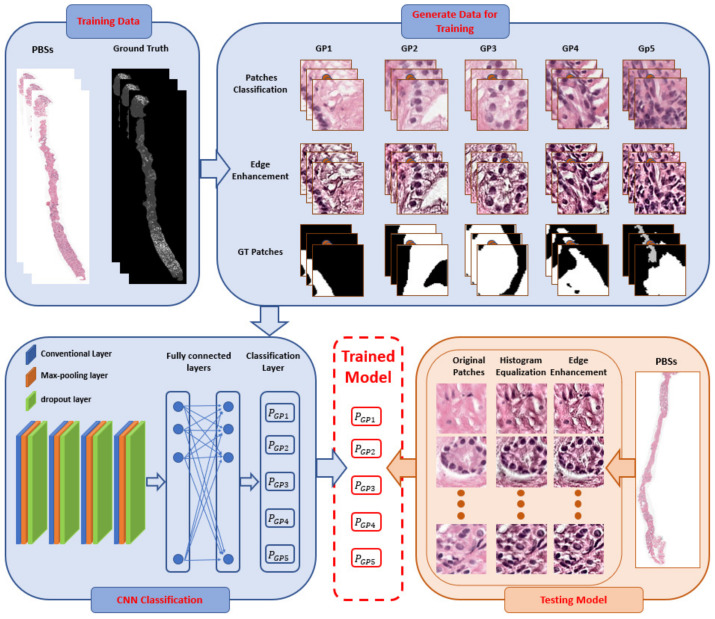
Proposed pipeline for patch-wise classification, while PBSs is prostate biopsy specimens and Gleason pattern labels (GP).

**Figure 4:**
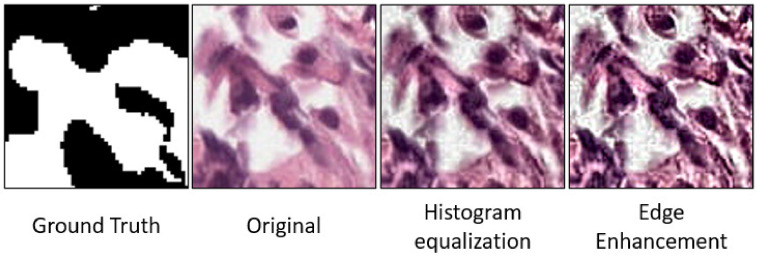
Example for applying prepossessing step on original patch: histogram equalization and edge enhancement.

The CNN plays an essential role in many fields of medical image analysis, especially in the segmentation [12,20,21], and classification [22,23]. Our DL system has a pyramidal architecture containing three CNNs, see Figure 2. The overall framework for our DL system is depicted in Figure 3, which shows the training and testing phases for for patch-wise classification. For the training model, the preprocessing step is applied to prepare the input data for the CNN. The preprocessing includes histogram equalization followed by edge enhancement [24,25]. The edge enhancement is applied to make the edges visible prominently by increasing the contrast of the pixels around the specific edges. The convolution matrix, namely, mask or kernel, is utilized to enhance the edges [24,25]. Figure 4 shows the effect of applying edge enhancement and histogram equalization on the original prostate patch. After that, the PBSs images are divided into overlapping patches with three different sizes: 100 × 100, 150 × 150, and 200 × 200 pixels, see Figure 2. The overlap between successive patches is 75%. The generation of overlapped patches provides different image viewpoints that enhance the DL framework’s training and validation. We select for training those patches with no more than 30% of their area labeled as background in the ground truth. Each patch is assigned a single label, being the ground truth GP of most pixels in the patch. If the winning label matches the value of the center of the given patch, then this patch is selected for training. Otherwise, we remove it from the CNN training. Algorithm 1 presents all details about the preprocessing step.

The pyramidal CNN is composed of three CNNs, and each one has a different patch size, as shown in Figure 2. We designed the three CNNs such that they have the same architecture but with different sizes. The prominent architecture of any DL-CNN base consists of input layers, hidden layers, and an output layer [26]. Our CNN’s input layer is fed with the patches from the first step (preprocessing) for our proposed framework. The small CNN ($CNN_{S}$) is fed with 100 × 100 patches, the medium CNN ($CNN_{M}$) is fed with 150 × 150 patches, and the large CNN ($CNN_{L}$) is provided with 200 × 200 patches. The core of CNN is the hidden layers that contain the number of CNN parameters and weights. The architecture of the hidden layers of our CNN is represented by a series of convolution layers (CLs) intervened by max-pooling layers (MPLs) and dropout layer, followed by two fully connected layers (FCls). Finally, there is a soft-max layer to give the probability for the five classes.
**Algorithm 1:** Preprocessing step of developing input data for a convolution neural network (CNN). Value of N is 100, 150, or 200. **Input:** Prostate biopsy specimens digitized. **Output:** Classified selected patches into Gleason pattern labels.
1.Apply the histogram equalization and edge enhancement on the PBSs.2.Divide the PBSs into overlapping patches, with size N × N pixels and 75% overlapping.3.Selecting appropriate patches for training
Calculate the majority voting for the pathological patch, WL ← Winning Label.Estimate two variables for corresponding label path, RB←ratio of Background and CV ←Center value.If (WL==CV)&(RB≤0.3) Select the patchElse Remove the patch


In the CL, the image is convolved with kernels (multiple kernels in each layer) to extract prominent features that describe the object of interest in the input patch; these features are called feature maps. Therefore, each CL results in a volume of feature maps. In our implementation, we use kernels of size 3 × 3. In MPLs, the spatial dimensions are reduced by a factor of two. Benefits are twofold: firstly, keeping only the most prominent features, discarding those less essential, and secondly, reducing computational cost and training time. In our implementation, the stride is equal to one for all layers.

The CNN contains four convolution layers, and the number of CLs filters is 16, 32, 64, and 128. The dropout layers weight 0.1, 0.1, 0.3, 0.3. The number of units for the FCLs is 64 and 512, respectively. The training seeks to minimize the cross-entropy loss between the predicted probabilities and the ground truth labels. The dropouts layers follow FCLs to minimize network overfitting, and the dropout rate is set to 0.15 for both layers. The total and trainable parameters for $CNN_{S}$, $CNN_{M}$, and $CNN_{L}$, are 264,421, 534,757, and 952,549, respectively, and there are no nontrainable parameters for all of them. The labeled patches are used to train the proposed CNN. During the training, the CNN uses iterative optimization to learn its weights to maximize the number of correctly classified samples during prediction.

**Figure 5:**
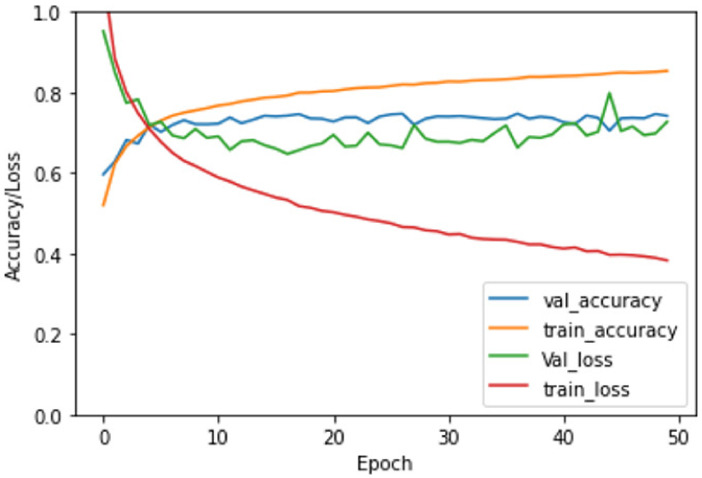
Training loss and accuracy as well as validation loss and accuracy in dataset for our proposed $CNN_{M}$.

Our DL model has numerous parameters. Therefore, we utilized a hyper-parameter tuning with a random search (RS) technique that helps to reduce overfitting and improves our model’s performance. Our system’s accuracy is assessed by performing training, validation, and testing for the patches. The curves for the accuracy and loss of training the $CNN_{L}$, the best accuracy among three CNN, are presented in Figure 5. Also, the validation (accuracy and loss) curves are shown in Figure 5. By increasing the number of epochs, the validation accuracy rises until it reaches around 0.78, and the validation loss decreases until it gets around 0.7.

### 2.2. Patch- and Pixel-Wise Classification

The goal for the patch-wise technique is to label all patches generated from the digitized PBS. We apply the patch-wise classification for all three CNNs individually during the test to assign each overlapped patch a label from one to five as GP categories. After that, a pixel-wise classification is applied to obtain three images of the same size as the original. The pixel-wise technique utilizes the output from the patch-wise classification, labeled patches generated from the three CNNs, to give all pixels that contain this patch the same label. Most of the pixels appear in several batches due to overlapped batches. Therefore, overlapped pixels have multiple labels depending on their position in the image. The majority voting is the core approach for the pixel-wise classification to get a single label for each overlapped pixel. The two techniques, patch- and pixel-wise, are used on the output of the three CNNs. The results after applying those techniques are three images of the same size as the original (each pixel has three labels); then, we applied majority voting again on those three images to obtain the final pixel-based classification.

### 2.3. Grade Groups System

The identification of the GG label is considered our goal in this work. Each digitized PBSs has labels between 1–5 according to its GP. We utilize Table 1 that demonstrates the relation between three measurements (GP, GS, and GG) to generate the GS from GP. Thus, identifying the GG from GS.

## 3. Results

The proposed framework is trained, validated, and tested on 416, 96, and 96, respectively, with whole slide images (WSIs) of the digitized PBSs from the Radboud University Medical Center, USA, and it was analyzed by the University of Louisville Hospital, USA [19,27]. A semiautomatic labeling technique was utilized to circumvent the need for full manual annotation by pathologists. Expert pathologists defined the GP, GS, and GG for all WSIs, and the digitized PBSs are divided into overlapping patches for patch and pixel-wise classification according to the GP ground truth. Our CAD software is primarily implemented in Python and Matlab programming environments. The experimental results were also performed on a Dell Precision workstation with an Intel Xeon eight-core CPU running at 3.31 GHz and 128 GB RAM.

The total number of patches for each $CNN_{S}$, $CNN_{M}$, and $CNN_{L}$ are around 5.8, 3.6, and 1.7 million, respectively. Those patches belong to 608 (416, 96, and 96) of the WSIs of the digitized PBS. The WSIs, 608, are separated into training, validation, and testing before generating the patches that means the model doesn’t see the validation or testing patches. We face a big challenge to train our model with a balanced dataset because the occurrence of the GP1 is very high with around 60% of the PBS, and the other four types (GP2, GP3, GP4, GP5) have almost 40%. Therefore, we utilized all batches generated from the four GP (GP2, GP3, GP4, GP5) during our training and randomly selected the number of patches from the GP1 that made the number of patches very close. The $CNN_{S}$, $CNN_{M}$, and $CNN_{L}$ were trained with around 130, 45, and 25,000 patches, respectively, for each group.

Our goal for this automated system is to identify the GG. Therefore, there are many steps, and each one has its results before reaching our target. We show first the accuracy for each CNN, pyramidal CNN, especially the patch-wise classification, see Table 2 and Table 3. After that, we demonstrate the system’s accuracy for the GG. More details are presented in the following two subsections.

### 3.1. Patch-Wise Classification for Each CNN

The overall diagnostic classification accuracy is evaluated using the accuracy metrics precision, recall, F1-score, accuracy, macro-averaged, and weighted-averaged [28,29]. The F1-score is computed by the formula:

(1) $$F1-score=\frac{2\times (precision\times recall)}{\left(precision+recall\right)}$$

The overall accuracy is the proportion of correctly classified samples out of all the samples; then, it must be equal for all metrics: precision, recall, F1-score. To summarize, the following always holds for the micro-F1.

(2) micro-F1=micro-precision=micro-recall=accuracy

since for the micro-averaging case, they are also equal to their harmonic mean; in other words, the micro-F1 case. The macro-average is also calculated for each metric and computed as simple arithmetic means of our per-class. The weighted average is the weighted of each class by the number of samples from that class.

The patch-wise classification results for our proposed pyramidal CNN are reported in Table 2 and Table 3. For $CNN_{S}$, $CNN_{M}$, and $CNN_{L}$, the classification accuracy is in the range 0.70–0.76. The macro-averaged and weighted-average metrics are found to be around 0.68–0.77. To highlight the advantages of using our DL (CNN), the accuracy of our pipeline is compared against standard ResNet50, and VGG-16 [22,30]. The input patches are resized into 224 × 224 pixels to fit with the image input size of the ResNet50 and VGG-16. The F1-score of VGG-16 is in the range of 0.58–0.70. In addition, the macro-averaged and weighted-average metrics are both found to be 0.63. The accuracy of our $CNN_{L}$ is high compared with that of the VGG-16 (0.65) and almost the same with that of ResNet50 (0.75), see Table 3. Besides the high accuracy for our CNN, there are two reasons for creating this CNN. Firstly, the computational cost for the RestNet50 and VGG-16 is high compared with that of our CNN. The number of parameters for ResNet50 and VGG-16 is almost 23 and 27 million, respectively, and our CNN is approximately one million. The second reason is that our pyramidal CNN needs a flexible size for the CNN, and a standard CNN like ResNet50 is a fixed-size that is 224 × 224.

### 3.2. Grade Group Results

**Figure 6:**
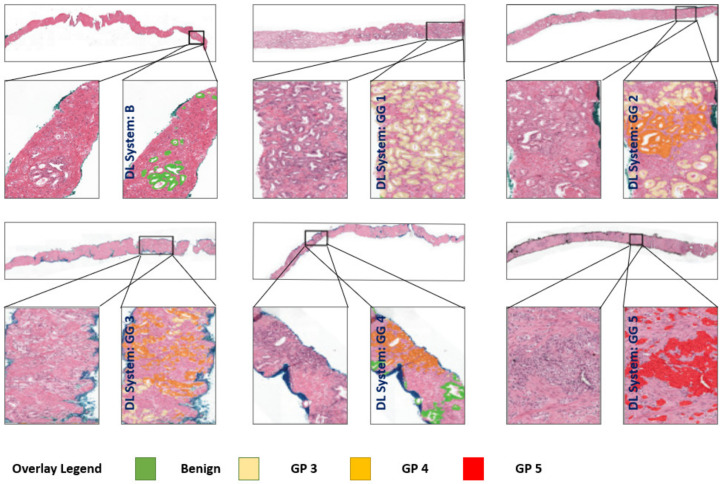
Examples of GG results from our system. In zoomed regions, system’s GP prediction is shown as an overlay on tissue; B (benign).

After applying the patch- and pixel-wise classification for each CNN, we merge the CNN outputs to obtain the production of the digitized PBS as the exact size of the original one. The new digitized PBS defines the results for GP from our automated system. We identify the GS and GG, the goal of our pipeline, using the fundamental converting that presents in the Table 1. Figure 6 shows the GG examples for our automated system results, which compare the reference standard and the predicted GG from our system using the distribution of GG.

**Figure 7:**
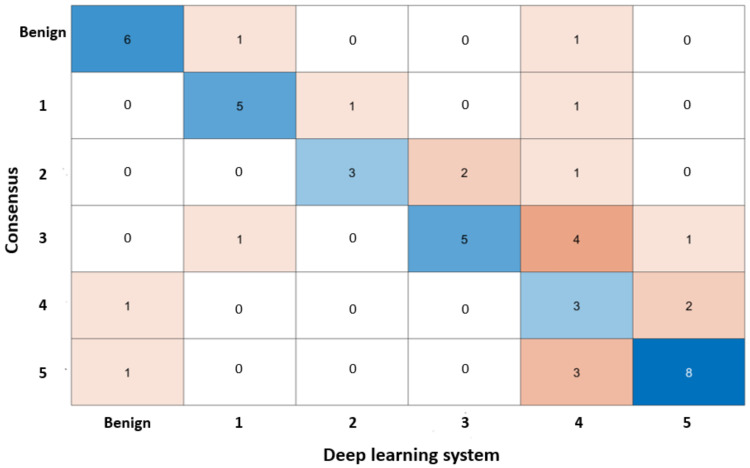
Confusion matrices on grade groups.

The overall GG diagnostic accuracy is summarized in Table 4, which presents the accuracy metrics precision, recall, F1-score, accuracy, and negative predictive value (NPV). Also, the confusion matrices for the grade groups results are shown in Figure 7. To validate our CAD system’s results and demonstrate its value, we compared our results with the pathologists’ estimated results, and previous work [19]. Firstly, the discordance for diagnosing the GG from the same biopsy is between 30% and 50% for various pathologists [3,7,8]. Therefore, our CAD system’s accuracy compared with that of pathologists’ estimated results is acceptable because our results are about 80% for precision, between 60–80% for recall and F1-score, and around 94% for accuracy and NPV. Secondly, the average accuracy and NPV for our automated CAD system to identify GG are 0.8767 and 0.9167, respectively, while the previous work [19] has 0.8500 and 0.9100, respectively. The obtained results show that our results compared with that of previous work are higher than two percent for an average of accuracy and almost the same for the NPV.

## 4. Discussion

The treatment for prostate cancer over the years improved for men with low-risk diseases. Notably, for patients with localized prostate cancer, active surveillance is safer compared with radical prostatectomy, as verified by many trials [31]. According to the American Society of Clinical Oncology, the GG and GP grading are considered the decision-maker according to the guideline of the American Society of Clinical Oncology [32]. The consults were recommended to enhance the consistency and quality of care due to the interobserver variability for the Gleason system [32,33]. Therefore, our automated CAD system could be a valuable decision support tool for patients’ GG with localized disease and give significant downstream treatment implications.

For that purpose, we developed an automated DL architecture for classifying the GG of digitized PBS. The ground truth of our datasets was performed using many experienced urologic subspecialists. They have around 25 years of experience with diverse backgrounds, and accessed several histologic sections and immunohistochemically stained sections for every specimen. The overall accuracy for our CAD system showed a similar rate compared with that of general pathologists, which is 70%. According to [34,35], the DL system can be used to alert pathologists of what might be missed. Otherwise, defining small tissue regions depends on a pathologist’s judgment that leads to overrule of false-positive categorizations. Therefore, our DL-CAD system has benefits for bolstering the selection of treatment modalities, especially for patients with localized disease.

Developing a framework with high accuracy is our ultimate goal in which DL, patch-, and pixel-wise classification were performed. The accuracy of the diagnostic results for the proposed framework using pyramidal CNN presents that $CNN_{L}$ had higher accuracy than that of $CNN_{S}$ and $CNN_{M}$, see Table 2 and Table 3 and Figure 5, which show the validation curves for the $CNN_{L}$. The comparison between the best CNN against the standard ResNet50 and VGG-16 [22,30], shown in Table 3, emphasizes the benefits of using the hyper-parameter tuning and the RS technique. Besides, using the new idea to identify the problem as classification one, not a segmentation, developed high accuracy.

The proposed CAD system can be helpful in healthcare systems in several ways, such as decreasing the consultation-associated costs, enhancing grading consistency, and reducing treatment-related morbidity for men with low-risk diseases. The performance metrics for GG estimation are higher for G1 and G2 grades compared with that of G3, G4, G5. Therefore, our automated system could be accurately classifying low-risk cases that are eligible for more conservative management.

The GG classification plays an essential role in prostate cancer treatment [31,36]. Still, it is not a straightforward task to the extent that there is no match the results between the subspecialists and the general pathologists’ for the GG classification. The subspecialists’ grading is more concordant than the general pathologists’ grading [37]. However, due to the difficulty of GG and inherent subjectivity, there is discordance between subspecialists. Therefore, it is critical to enhance the risk stratification for prostate cancer by overcoming those disagreements. Developing a system with high precision that human graders and predict clinical risk is our priority. Machine learning, especially DL, models could distinguish novel histoprognostic signals that the human eye can not discover [38,39], as well as assistance in stratifying patient risk like existing molecular tests [40].

Despite the promising results, our automated DL system has some limitations. Firstly, our DL model was trained and tested from a single institution. Therefore, using an external test dataset for the different centers and WSI with various staining protocols should further enhance the robustness of our automated system. Secondly, our DL models, as well the pathologists who made the ground truth labeling, treated each biopsy as an independent sample. In clinical practice, multiple biopsies are sampled from various regions of the prostate. Therefore, an update to our model could take multiple biopsies into account and give a grade group prediction at the patient level. Finally, our study concentrated on the grading of acinar adenocarcinoma in prostate biopsies. However, prostate biopsies can contain other tumor types and foreign tissue, such as colon glands. The biopsies could also include additional prognostic information, such as the detection of intraductal carcinoma [41].

## 5. Conclusions

This paper introduced a Deep Learning-based CAD system to classify the grade groups (GG) system using digitized prostate biopsy specimens (PBSs) using pyramidal CNN, with patch- and pixel-wise classifications. The proposed pipeline results highlight our system’s potential to classify all five GG of the PBSs in comparison with that of other standard CNNs. The agreement between our CAD system and pathologists is comparable to inter-rater reliability among pathologists. In future work, because the digitized PBSs do not have the same direction, adding a new preprocessing step to overcome this challenge will fit our results. This processing rotates the overlapped patches with angles 45 and 90, and flipping them will enhance our pyramidal CNN accuracy. In addition, to highlight our model, we will try to test our model with a dataset from another institution.

## Figures and Tables

**Table 1:** Grading systems for a prostate biopsy specimen are the Gleason grading system, the Gleason pattern (GP) and Gleason score (GS), as well as the grade groups (GP) system.

Shape of Cell Tissues		GP	Risk Level	GS	GG
stroma (connective tissue, non-epithelium tissue)		GP1	-	-	-
healthy (benign) epithelium		GP2	-	-	-
moderately differentiated	Distinctly infiltration of cells form glands at margins	GP3	Low	GP3 + GP3 = GS6	GG1
			Favorable	GP3 + GP4 = GS7	GG2
moderately and Poorly differentiated	Irregular messes of neoplastic cells with few glands	GP4	Unfavorable	GP4 + GP3 = GS7	GG3
			High	GP4 + GP4 = GS8	GG4
				GP3 + GP5 = GS8	
				GP5 + GP3 = GS8	
Poorly differentiated	Lack of or occasional glands, sheets of cells	GP5	High	GP4 + GP5 = GS9	GG5
				GP5 + GP4 = GS9	
				GP5 + GP5 = GS10	

**Table 2:** Patch-wise classification accuracy for our proposed $CNN_{S}$ and $CNN_{L}$ using precision, recall, F1-score, accuracy, macro-averaged, and weighted-averaged.

	${\mathrm{CNN}}_{S}$			${\mathrm{CNN}}_{M}$			${\mathrm{CNN}}_{L}$		
	Precision	Recall	F1-Score	Precision	Recall	F1-Score	Precision	Recall	F1-Score
**Class 1**	0.86	0.97	0.91	0.92	0.97	0.94	0.96	0.94	0.95
**Class2**	0.74	0.79	0.76	0.77	0.80	0.78	0.81	0.82	0.81
**Class3**	0.66	0.63	0.76	0.68	0.62	0.65	0.75	0.68	0.71
**Class4**	0.66	0.44	0.52	0.63	0.55	0.59	0.64	0.66	0.65
**Class5**	0.53	0.66	0.59	0.46	0.63	0.53	0.42	0.53	0.47
**Accuracy**	0.70	0.70	0.70	0.73	0.73	0.73	0.76	0.76	0.76
**Macro-averaged**	0.68	0.70	0.69	0.69	0.71	0.70	0.72	0.73	0.72
**Weighted-average**	0.70	0.70	0.70	0.73	0.73	0.72	0.77	0.76	0.76

**Table 3:** Patch-wise classification accuracy for our proposed $CNN_{L}$ and VGG-16 network using precision, recall, F1-score, accuracy, macro-averaged, and weighted-averaged.

	${\mathrm{CNN}}_{L}$			VGG-16			ResNet50		
	Precision	Recall	F1-Score	Precision	Recall	F1-Score	Precision	Recall	F1-Score
**Class1**	0.96	0.94	0.95	0.87	0.82	0.85	0.94	0.93	0.94
**Class2**	0.81	0.82	0.81	0.76	0.56	0.64	0.74	0.93	0.82
**Class3**	0.75	0.68	0.71	0.65	0.58	0.62	0.75	0.65	0.70
**Class4**	0.64	0.66	0.65	0.51	0.63	0.56	0.72	0.54	0.61
**Class5**	0.42	0.53	0.47	0.35	0.59	0.44	0.58	0.76	0.66
**Accuracy**	0.76	0.76	0.76	0.65	0.65	0.65	0.75	0.75	0.75
**Macro-averaged**	0.72	0.73	0.72	0.63	0.64	0.62	0.75	0.76	0.75
**Weighted-average**	0.77	0.76	0.76	0.68	0.65	0.65	0.77	0.76	0.76

**Table 4:** Grade groups classification of our CAD system using precision, recall, F1-score, accuracy, and negative predictive value (NPV).

	Precision	Recall	F1-Score	Accuracy	NPV	Cases
**Benign**	0.75	0.75	0.76	0.92	0.95	8
**GG1**	0.71	0.71	0.71	0.92	0.95	7
**GG2**	0.75	0.50	0.60	0.92	0.93	6
**GG3**	0.71	0.45	0.56	0.84	0.86	11
**GG4**	0.23	0.50	0.32	0.80	0.92	6
**GG5**	0.73	0.67	0.70	0.86	0.89	12

## Data Availability

The data used in this study is available from https://www.kaggle.com/c/prostate-cancer-grade-assessment, accessed on 3 October 2021.

## References

[1] American Cancer Society (2021). Cancer Facts and Figures. https://www.cancer.org/research/cancer-facts-statistics/all-cancer-facts-figures/cancer-facts-figures-2021.html.

[2] Litwin M.S., Tan H.J. (2017). The diagnosis and treatment of prostate cancer: A review. JAMA.

[3] Veloso S.G., Lima M.F., Salles P.G., Berenstein C.K., Scalon J.D., Bambirra E.A. (2007). Interobserver agreement of Gleason score and modified Gleason score in needle biopsy and in surgical specimen of prostate cancer. Int. Braz. J. Urol..

[4] Nagpal K., Foote D., Liu Y., Chen P.H.C., Wulczyn E., Tan F., Olson N., Smith J.L., Mohtashamian A., Wren J.H. (2019). Development and validation of a deep learning algorithm for improving Gleason scoring of prostate cancer. NPJ Digit. Med..

[5] Matoso A., Epstein J.I. (2019). Defining clinically significant prostate cancer on the basis of pathological findings. Histopathology.

[6] Epstein J.I., Egevad L., Amin M.B., Delahunt B., Srigley J.R., Humphrey P.A. (2016). The 2014 International Society of Urological Pathology (ISUP) consensus conference on Gleason grading of prostatic carcinoma. Am. J. Surg. Pathol..

[7] Melia J., Moseley R., Ball R., Griffiths D., Grigor K., Harnden P., Jarmulowicz M., McWilliam L., Montironi R., Waller M. (2006). A UK-based investigation of inter-and intra-observer reproducibility of Gleason grading of prostatic biopsies. Histopathology.

[8] Egevad L., Ahmad A.S., Algaba F., Berney D.M., Boccon-Gibod L., Compérat E., Evans A.J., Griffiths D., Grobholz R., Kristiansen G. (2013). Standardization of Gleason grading among 337 European pathologists. Histopathology.

[9] Kvåle R., Møller B., Wahlqvist R., Fosså S.D., Berner A., Busch C., Kyrdalen A.E., Svindland A., Viset T., Halvorsen O.J. (2009). Concordance between Gleason scores of needle biopsies and radical prostatectomy specimens: A population-based study. BJU Int..

[10] Bottke D., Golz R., Störkel S., Hinke A., Siegmann A., Hertle L., Miller K., Hinkelbein W., Wiegel T. (2013). Phase 3 study of adjuvant radiotherapy versus wait and see in pT3 prostate cancer: Impact of pathology review on analysis. Eur. Urol..

[11] Kasivisvanathan V., Rannikko A.S., Borghi M., Panebianco V., Mynderse L.A., Vaarala M.H., Briganti A., Budäus L., Hellawell G., Hindley R.G. (2018). MRI-targeted or standard biopsy for prostate-cancer diagnosis. N. Engl. J. Med..

[12] Hammouda K., Khalifa F., Soliman A., Abdeltawab H., Ghazal M., Abou El-Ghar M., Haddad A., Darwish H.E., Keynton R., El-Baz A. A 3D CNN with a Learnable Adaptive Shape Prior for Accurate Segmentation of Bladder Wall Using MR Images. Proceedings of the 2020 IEEE 17th International Symposium on Biomedical Imaging (ISBI).

[13] Wildeboer R.R., van Sloun R.J., Wijkstra H., Mischi M. (2020). Artificial intelligence in multiparametric prostate cancer imaging with focus on deep-learning methods. Comput. Methods Programs Biomed..

[14] Hammouda K., Khalifa F., Soliman A., Ghazal M., Abou El-Ghar M., Badawy M., Darwish H., Khelifi A., El-Baz A. (2021). A multiparametric MRI-based CAD system for accurate diagnosis of bladder cancer staging. Comput. Med. Imaging Graph..

[15] Reda I., Khalil A., Elmogy M., Abou El-Fetouh A., Shalaby A., Abou El-Ghar M., Elmaghraby A., Ghazal M., El-Baz A. (2018). Deep learning role in early diagnosis of prostate cancer. Technol. Cancer Res. Treat..

[16] Schelb P., Kohl S., Radtke J.P., Wiesenfarth M., Kickingereder P., Bickelhaupt S., Kuder T.A., Stenzinger A., Hohenfellner M., Schlemmer H.P. (2019). Classification of cancer at prostate MRI: Deep learning versus clinical PI-RADS assessment. Radiology.

[17] Mehrtash A., Sedghi A., Ghafoorian M., Taghipour M., Tempany C.M., Wells W.M., Kapur T., Mousavi P., Abolmaesumi P., Fedorov A. (2017). Classification of clinical significance of MRI prostate findings using 3D convolutional neural networks. Proceedings of the Medical Imaging 2017: Computer-Aided Diagnosis.

[18] Arvaniti E., Fricker K.S., Moret M., Rupp N., Hermanns T., Fankhauser C., Wey N., Wild P.J., Rueschoff J.H., Claassen M. (2018). Automated Gleason grading of prostate cancer tissue microarrays via deep learning. Sci. Rep..

[19] Bulten W., Pinckaers H., van Boven H., Vink R., de Bel T., van Ginneken B., van der Laak J., de Kaa C.H.v., Litjens G. (2019). Automated gleason grading of prostate biopsies using deep learning. arXiv.

[20] Hammouda K., Khalifa F., Abdeltawab H., Elnakib A., Giridharan G., Zhu M., Ng C., Dassanayaka S., Kong M., Darwish H. (2020). A new framework for performing cardiac Strain Analysis from cine MRi imaging in Mice. Sci. Rep..

[21] Kamnitsas K., Ledig C., Newcombe V.F., Simpson J.P., Kane A.D., Menon D.K., Rueckert D., Glocker B. (2017). Efficient multi-scale 3D CNN with fully connected CRF for accurate brain lesion segmentation. Med Image Anal..

[22] Russakovsky O., Deng J., Su H., Krause J., Satheesh S., Ma S., Huang Z., Karpathy A., Khosla A., Bernstein M. (2015). Imagenet large scale visual recognition challenge. Int. J. Comput. Vis..

[23] Krizhevsky A., Sutskever I., Hinton G.E. (2012). Imagenet classification with deep convolutional neural networks. Adv. Neural Inf. Process. Syst..

[24] Kaur M., Kaur J., Kaur J. (2011). Survey of contrast enhancement techniques based on histogram equalization. Int. J. Adv. Comput. Sci. Appl..

[25] Nnolim U.A. (2017). Smoothing and enhancement algorithms for underwater images based on partial differential equations. J. Electron. Imaging.

[26] Shin H.C., Roth H.R., Gao M., Lu L., Xu Z., Nogues I., Yao J., Mollura D., Summers R.M. (2016). Deep convolutional neural networks for computer-aided detection: CNN architectures, dataset characteristics and transfer learning. IEEE Trans. Med Imaging.

[27] (2021). Website for the Dataset. https://www.kaggle.com/c/prostate-cancer-grade-assessment.

[28] Hossin M., Sulaiman M.N. (2015). A review on evaluation metrics for data classification evaluations. Int. J. Data Min. Knowl. Manag. Process..

[29] McNee S.M., Riedl J., Konstan J.A. Being accurate is not enough: How accuracy metrics have hurt recommender systems. Proceedings of the CHI’06 Extended Abstracts on Human Factors in Computing Systems.

[30] He K., Zhang X., Ren S., Sun J. Deep residual learning for image recognition. Proceedings of the IEEE Conference on Computer Vision and Pattern Recognition.

[31] Lane J.A., Donovan J.L., Davis M., Walsh E., Dedman D., Down L., Turner E.L., Mason M.D., Metcalfe C., Peters T.J. (2014). Active monitoring, radical prostatectomy, or radiotherapy for localised prostate cancer: Study design and diagnostic and baseline results of the ProtecT randomised phase 3 trial. Lancet Oncol..

[32] Chen R.C., Rumble R.B., Loblaw D.A., Finelli A., Ehdaie B., Cooperberg M.R., Morgan S.C., Tyldesley S., Haluschak J.J., Tan W. (2016). Active surveillance for the management of localized prostate cancer (Cancer Care Ontario guideline): American Society of Clinical Oncology clinical practice guideline endorsement. J. Clin. Oncol. Off. J. Am. Soc. Clin. Oncol..

[33] Brimo F., Schultz L., Epstein J.I. (2010). The value of mandatory second opinion pathology review of prostate needle biopsy interpretation before radical prostatectomy. J. Urol..

[34] Steiner D.F., MacDonald R., Liu Y., Truszkowski P., Hipp J.D., Gammage C., Thng F., Peng L., Stumpe M.C. (2018). Impact of deep learning assistance on the histopathologic review of lymph nodes for metastatic breast cancer. Am. J. Surg. Pathol..

[35] Liu Y., Kohlberger T., Norouzi M., Dahl G.E., Smith J.L., Mohtashamian A., Olson N., Peng L.H., Hipp J.D., Stumpe M.C. (2019). Artificial intelligence–based breast cancer nodal metastasis detection: Insights into the black box for pathologists. Arch. Pathol. Lab. Med..

[36] Gislén A., Dacke M., Kröger R.H., Abrahamsson M., Nilsson D.E., Warrant E.J. (2003). Superior underwater vision in a human population of sea gypsies. Curr. Biol..

[37] Allsbrook W.C., Mangold K.A., Johnson M.H., Lane R.B., Lane C.G., Epstein J.I. (2001). Interobserver reproducibility of Gleason grading of prostatic carcinoma: General pathologist. Hum. Pathol..

[38] Courtiol P., Maussion C., Moarii M., Pronier E., Pilcer S., Sefta M., Manceron P., Toldo S., Zaslavskiy M., Le Stang N. (2019). Deep learning-based classification of mesothelioma improves prediction of patient outcome. Nat. Med..

[39] Wulczyn E., Steiner D.F., Xu Z., Sadhwani A., Wang H., Flament-Auvigne I., Mermel C.H., Chen P.H.C., Liu Y., Stumpe M.C. (2020). Deep learning-based survival prediction for multiple cancer types using histopathology images. PLoS ONE.

[40] Marrone M., Potosky A.L., Penson D., Freedman A.N. (2015). A 22 gene-expression assay, Decipher®(GenomeDx Biosciences) to predict five-year risk of metastatic prostate cancer in men treated with radical prostatectomy. PLoS Curr..

[41] Kweldam C.F., Kümmerlin I.P., Nieboer D., Verhoef E.I., Steyerberg E.W., Van der Kwast T.H., Roobol M.J., van Leenders G.J. (2016). Disease-specific survival of patients with invasive cribriform and intraductal prostate cancer at diagnostic biopsy. Mod. Pathol..

